# Filter paper performance in PCR for cutaneous leishmaniasis diagnosis

**DOI:** 10.1590/0037-8682-0047-2020

**Published:** 2020-12-21

**Authors:** Camila Alves Mota, Eneide Aparecida Sabaini Venazzi, Paulo Donizeti Zanzarini, Sandra Mara Alessi Aristides, Maria Valdrinez Campana Lonardoni, Thaís Gomes Verzignassi Silveira

**Affiliations:** 1 Universidade Estadual de Maringá, Programa de Pós-Graduação em Biociências e Fisiopatologia, Maringá, PR, Brasil.; 2 Universidade Estadual de Maringá, Departamento de Análises Clínicas e Biomedicina, Laboratório de Parasitologia Clínica, Maringá, PR, Brasil.; 3 Universidade Estadual de Maringá, Departamento de Análises Clínicas e Biomedicina, Laboratório de Leishmanioses, Maringá, PR, Brasil.

**Keywords:** Leishmania, Cutaneous leishmaniasis, Neglected diseases, Polymerase chain reaction, Diagnosis

## Abstract

**INTRODUCTION:**

The objective of this study was to evaluate the performance of filter paper (FP) for lesion scraping collection in a polymerase chain reaction (PCR) for cutaneous leishmaniasis (CL) diagnosis.

**METHODS:**

Lesion scrapings from 48 patients were collected and analyzed for PCR.

**RESULTS:**

PCR with FP detected up to three *Leishmania braziliensis* promastigotes. Considering the direct search by microscopy or PCR of samples collected in STE buffer as standards, the sensitivity of PCR with FP was 100%.

**CONCLUSIONS:**

FP can be useful for CL diagnosis in remote regions, allowing high sensitivity in the detection of the parasite by PCR.

Cutaneous leishmaniasis (CL) is a neglected disease and significant public health problem. In the last five years, one million cases of CL were reported worldwide, and more than 90% of them occurred in Afghanistan, Algeria, Brazil, Colombia, Iran, Pakistan, Peru, Saudi Arabia, and Syria^1^. 

Collecting material from CL lesions by biopsy is invasive and demands extra care when it comes to sample preservation^2^. A precise molecular diagnosis depends on obtaining adequate samples and also on DNA integrity^3^. Filter paper (FP) is an important tool for collecting, storing and transporting samples for the diagnosis of neglected diseases^4^. 

Considering the importance of accurate CL diagnosis, especially for populations living in regions which are difficult to access, the objective of this study was to evaluate the performance of FP for lesion scraping collection, including the sensitivity of the test and the number of *Leishmania* parasites that can be detected on the FP with the purified material used directly in PCR. 

To determine the sensitivity of PCR with FP, lesion scrapings were collected from CL-suspected patients referred to the Laboratório de Ensino e Pesquisa em Análises Clínicas (LEPAC) of the State University of Maringá (UEM) for CL diagnosis. These patients lived in municipalities belonging to the North-Central Paraná Mesoregion, Brazil, which is endemic for CL ( Supplementary Figure 1). The patients were informed about the project and signed an Informed Consent Form. This study followed resolution number 466/2012-CNS of the National Health Council of the Health Ministry (Brazil) and the Helsinki Declaration from 1975. It was approved (ethical approval number:865.567/2014) by the Permanent Committee on Ethics in Research Involving Human Beings of UEM. Patients who had at least one positive result in direct search (DS) by microscopy of Giemsa-stained smear or the Montenegro skin test (MST) were considered CL cases. 

Samples were collected by scraping of the lesion’s inner edge. For DS, lesion scrapings were placed on glass slides, stained with Giemsa, and examined by microscopy for *Leishmania* sp. amastigotes. The lesion scrapings were also distributed into microtubes containing 50 μL of sodium chloride-Tris-EDTA (STE) buffer (10 mM Tris, 1mM Na_2_EDTA∙H_2_O; 0.1 M NaCl, pH 8.0) and stored at -20 ºC for further PCR testing. The DNA was obtained by incubation at 95 ºC for 30 min in a Veriti^®^ Thermocycler (Applied Biosystems^®^, USA) followed by centrifugation at 13.000 *g* for 1 min. The supernatant containing the DNA was precipitated and stored at 4 ºC. 

Lesion scrapings were also placed on FP (Whatman^®^ FTA^TM^ Classic card, GE Healthcare, UK) and stored in plastic bags at room temperature. A disk (d = 2 mm) was cut out of the FP using an FTA^TM^ Harris Micro-Punch instrument and transferred to a PCR microtube. The total DNA of the lesion scraping contained in the disk was purified with 200 μL of FTA^TM^ Purification Reagent (Amersham Buckinghamshire, UK) according to the manufacturer’s instructions (Figure 1).

**FIGURE 1:**
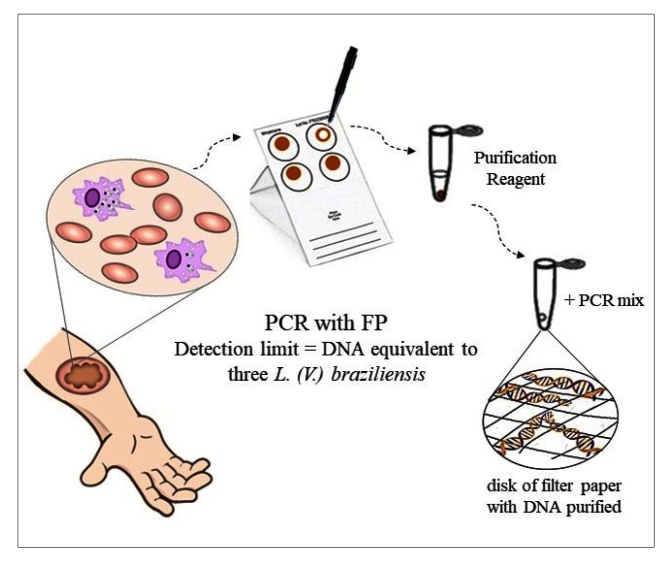
Illustrative flow showing the lesion scraping collection through the performance of PCR with FP.

MST was carried out by intradermal injection of 0.1 mL of antigen into the patient’s arm. The test result was verified after 48 h and an induration ≥ 5 mm in diameter was considered positive.

PCR was performed using the primers MP3H (5′-GAACGGGGTTTCTGTATGC-3′) and MP1L (5′-TACTCCCCGACATGCCTCTG-3′)^5^, which amplify a 70 bp fragment of *Leishmania (Viannia)* kinetoplast DNA (kDNA). The reaction mixture (25 μL) was composed of 1 μM of primers (Invitrogen, Brazil), 0.2 mM dNTP (Invitrogen, USA), 1U Taq DNA Polymerase (Invitrogen, USA), 1.5 mM MgCl_2_, 1X Enzyme Buffer, and 5 μL of DNA obtained from lesion scraping collected in a microtube with STE. Alternatively, 25 μL of the reaction mixture was added directly to the microtube containing the disk of FP with the purified lesion scraping sample^6^. 

DNA amplification was performed with a Veriti^TM^ 96-Well Thermal Cycler (Applied Biosystems, USA) at 95 °C for 5 min, followed by 30 cycles, each one divided into denaturation (95 °C, 1.5 min), annealing (56 °C, 1.5 min), and elongation (72 °C, 2 min), plus a final elongation at 72 °C for 10 min. The amplified products were subjected to electrophoresis in 3% agarose gel, and the bands revealed with 0.1 μg/mL ethidium bromide in a transilluminator (LTB-20X20 HE, Loccus Biotecnologia, Brazil).

FP positive control was 5 ng of *L. (V.) braziliensis* DNA placed on a disk (d = 2 mm) and treated in the same way as the samples. FP negative control consisted of one disk of FP, not subjected to Purification Reagent, to evaluate if the FTA^TM^ HarrisMicro-Punch instrument causes a false-positive result. Another disk was subjected to Purification Reagent, to assess if the reagent was free of contamination. Positive (5 ng of *L. (V.) braziliensis* DNA) and negative (ultrapure water) amplification controls were also included in all PCR carried out.

For performance assays, a serial dilution of *L. (V.) braziliensis* promastigotes was used to measure FP’s capacity to retain and preserve *Leishmania* DNA and to determine the minimal number of *Leishmania* parasites per punch that are required for successful PCR amplification. Assay 1 was performed with *L. (V.) braziliensis* promastigotes (3x10^5^ to 3x10^-3^/μL) suspended in PBS and spotted on FP to determine the detection limit of the test for the parasite alone. Assay 2 was a mixture of lesion scrapings from patients with negative CL diagnosis plus 3x10^5^ to 3x10^-3^ *L. (V.) braziliensis* promastigotes/µL serial dilution, to investigate interference of non-target DNA (human genetic material) in the detection of parasite DNA in lesion samples collected on FP. Volumes of 1 μL from each dilution were deposited on 2 mm disks of FP, treated in the same way as the samples, and submitted directly to the PCR reaction mixture. A control, not spotted on FP, with the dilutions of DNA equivalent to 3x10^5^ to 3x10^-3^ *L. (V.) braziliensis* promastigotes, was made in parallel.

The data were analyzed by McNemar’s test and Cochrane’s Q test using the BioEstat 5.3, and the Screening test using OpenEpi version 2.3.1, with a significance level of 5%. The parameters specificity (E) and sensitivity (S) were determined in relation to DS and PCR with samples collected in STE.

The PCR of assay 1 showed a 70 bp band until the sixth dilution point, so it was able to amplify the DNA from three *L. (V.) braziliensis* promastigotes spotted on a 2 mm disk of FP (Figure 2A). Assay 2, like assay 1, exhibited a 70 bp band up to the dilution point corresponding to three *L. (V.) braziliensis* promastigotes/µL in lesion cell suspension spotted on FP (Figure 2B). The control assay amplified the DNA equivalent to 0.3 *L. (V.) braziliensis* promastigotes (approximately 25 fg), without being placed on FP (Figure 2C).

**FIGURE 2:**
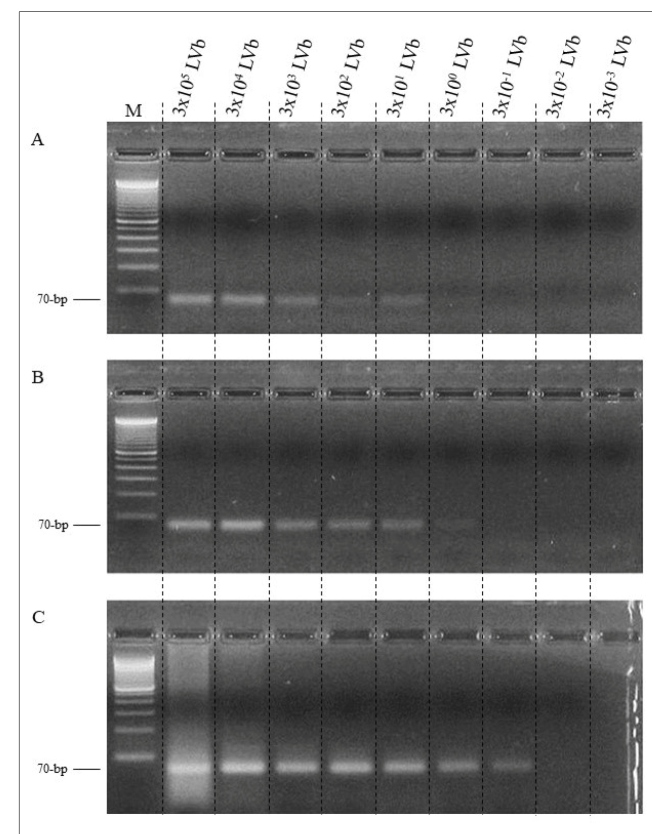
Polymerase chain reaction in 3% agarose gel showing 70 bp fragments from the kDNA minicircle region of *Leishmania (Viannia)* to evaluate filter paper performance. **A**: Assay 1 (PCR with FP), lanes 1 to 9, serial dilution ranging from 3x10^5^ to 3x10^-3^ *L. (V.) braziliensis* (LVb) promastigotes suspended in PBS, spotted on filter paper. **B**: Assay 2 (PCR with FP), lanes 1 to 9, a serial dilution of cells from lesions negative for CL plus 3x10^5^ to 3x10^-3^ LVb promastigotes/µL. M: 100-bp molecular weight. **C**: Control assay (PCR without FP), lanes 1 to 9, DNA equivalent of 3x10^5^ to 3x10^-3^ promastigotes. M: 100 bp molecular weight.

Lesion samples from 48 Brazilian patients living in CL endemic regions of Paraná state were analyzed. The patients’ average age was 47 years (range, 18-68 years); most of them were men (69%; 33/48). The main place where they were infected was the rural area (75%; 36/48). The patients had a single lesion predominance (69%; 33/48). The evolution time of the primary lesions ranged from one week to twenty-four months.

Among the patients, 48% (23/48) [95% CI; 34.9-63.1] were DS positive. The positivity of PCR with lesion scrapings collected in STE was 50% (24/48) [95% CI; 36.1-63.9] and had no difference from DS (*P* > 0.05, McNemar’s test). PCR of FP showed a positivity of 52% (25/48) [95% CI; 38-65.9], similar to DS (*P* > 0.05, McNemar’s test) and PCR of samples collected in STE (*P* > 0.05, McNemar’s test).

For the 26 patients with CL diagnosis by DS and/or MST, the positivity for PCR of lesion scrapings collected in STE was 92% (24/26) [95% CI; 76.8-98.7], and for PCR of lesion scrapings in FP was 96% (25/26) [95% CI; 82.5-99.8). The positivity for PCR of samples collected in STE was no different from DS (*P* > 0.05, McNemar’s test); PCR with FP presented positivity similar to DS (*P* > 0.05, McNemar’s test) and PCR of samples collected in STE (*P >* 0.05, McNemar’s test). PCR with FP was positive in 100% (23/23) [95% CI; 87.8-100] of patients with DS positivity and 8% (2/25) [95% CI; 1.3-24] of those found negative by DS. 

Compared to DS or PCR of samples collected in STE, the sensitivity of PCR with FP according to the Screening test was 100% [95% CI, 85.7-100; 86.2-100] for both, and its specificity was 92% [95% CI; 75-97.8] and 96% [95% CI; 79.8-99.3], respectively. For patients who received CL diagnosis by DS and/or MST, the sensitivity and specificity of PCR with FP were 96% [95% CI; 81.1-99.3] and 95% [95% CI; 78.2-99.2], respectively (Table 1). Analysis showed that the three methods, DS and PCR with STE or FP, are equally good for detecting the *Leishmania* parasite in lesion scrapings (*P >* 0.05, Cochrane’s Q test).

**TABLE 1:** Performance of PCR with FP sampling method compared to tests involving direct search by microscopy and PCR with samples collected in STE, and to CL diagnosed patients.

PCR with FP	Direct search of parasite		PCR with STE		Positive CL cases*	
	Positive	Negative	Positive	Negative	Positive	Negative
	(n=23)	(n=25)	(n=24)	(n=24)	(n=26)	(n=22)
Positive (n=25)	23	2	24	1	25	1
Negative (n=23)	0	23	0	23	1	21
Sensitivity (%; 95% CI)	100; (85.7-100)		100; (86.2-100)		96; (81.1-99.3)	
Specificity (%; 95% CI)	92; (75-97.8)		96; (79.7-99.3)		95; (78.2-99.2)	

*Patients who had positive results by direct search of *Leishmania* and/or Montenegro skin test were considered to have positive CL diagnosis.

In this study, we assessed FP performance, encompassing its capacity to preserve DNA and to be used directly in PCR for CL diagnosis^6^. According to our search of the scientific literature, some studies have already evaluated the sensitivity of PCR with samples collected on FP for CL diagnosis^6^ ^-^ ^8^, but no work has shown the detection limit of PCR with the direct use of FP containing the *Leishmania* DNA as we did in our research, i.e., in a serial dilution placed on the paper. Also, the primer chosen has already been applied in several studies involving DNA detection of *Leishmania (Viannia)* ^9^ ^,^ ^10^. Assay 2 was done to evaluate the interference of non-target DNA in the detection of *Leishmania* from lesion samples collected on FP and was constructed in a way that imitates real biological conditions to the maximum extent. In this assay, it was possible to detect up to three promastigotes on a single punched disk, equivalent to 0.25 pg of DNA, considering the sequenced genome of *L. (V.) braziliensis* (MHOM/BR/75/M2904)^9^. The intensity of the amplicon band of assay 2 corresponding to three *Leishmania* parasites, was weak compared to the band of assay 1 that amplified the same quantity of parasites. When comparing assay 2 with the control dilution, a 10-fold difference in the number of detected parasites was observed. These differences are related to the interference of non-target DNA, carried with the patient’s cells. For this reason, high sensitivity primers should be chosen to perform PCRs with FP.

Santos (2012) shows that the direct use of the FTA^TM^ elute card in the mix was more effective in identifying HPV-DNA (92% of positive samples) than DNA extracted from the card by elution (54%)^11^. Studies involving *Leishmania* that used FP for the collection of lesion scrapings, biopsy, or lesion imprint had greater sensitivity (92% to 100%) compared to DS or PCR with DNA obtained from lesion aspirate or biopsy. These results were obtained either by extracting the DNA from the card or by using it directly in the PCR mixture^6^ ^,^ ^12^. FP does not appear to interfere with DNA amplification. The increase in temperature of the PCR mix was probably enough for the DNA to escape the FP, thus allowing an efficient reaction^11^.

When tested with clinical specimens from CL-suspected patients, the PCR with FP was positive for two patients with negative CL diagnosis by DS, which was fortunate for these people. Still, most likely due to the number of patients analyzed, this difference in positivity was not statistically significant. There is a reduction in the number of parasites in the lesion with the progression of the disease, and DS (by microscopy) sensitivity can be limited by the expertise of the laboratory technician who performs the test^13^. Using microscopy, culture, and PCR methods, Eroglu (2014) evaluated the sensitivities of skin samples taken in smear, aspiration fluid, and FP for CL diagnosis in the Old World^14^. Using similar techniques to those in the previously cited study, Al-Jawabreh (2018) compared unstained smears, smears stained with Giemsa stain, and FP, using microscope, culture, and PCR for CL diagnosis. In both of these studies, the lesion samples collected on FP revealed the most CL cases^8^.

The sensitivity of PCR using FP was 100% compared to both DS and PCR with lesion scrapings collected in STE, and specificity was 92% and 96%, respectively. This small decrease in specificity can be explained by the test results of one patient who was DS negative but positive for both types of PCR (STE and FP), and another who was showed negative using DS and PCR with STE, but positive with PCR using FP. The method of specimen collection affects DNA yield and, consequently, the test’s sensitivity^7^ ^,^ ^8^. Samples collected on FP may have a higher capacity to preserve the DNA^7^ ^,^ ^13^. 

This study had a limitation due to the lack of a control group of healthy patients, due to the impossibility of collecting biological material from these patients. For this reason, lesions from patients with other dermatologic diseases who had a negative diagnosis for CL were considered as a control group. 

We also suggest the use of a less invasive and painful collection method, such as scraping instead of a biopsy, for the search of *Leishmania* in patients with cutaneous lesions^2^. FP preserves the biological material, when it is properly collected, and its association with a sensitive technique such as PCR can contribute significantly to disease diagnosis and appropriate treatment. In particular, it can be applied in cases of individuals who live in difficult-to-access areas, where preservation of the collected material becomes precarious. FP is a safe and easy collecting tool that avoids contamination risks. It allows preservation of material obtained by a less invasive method, such as lesion scraping or imprinting, and allows quick and effective DNA collection^6^ ^,^ ^7^. It also provides a functional way to transport the samples, and can be stored at room temperature for extended periods^15^. This study showed that it is possible to detect up to three *Leishmania* parasites in lesion scrapings spotted on FP with a single punched disk, directly used in the PCR amplification mix. FP may be an alternative tool, with high performance, to preserve samples for field collection, especially in difficult-to-access regions, where, until recently, CL has caused stigma and affected the most impoverished populations.
