# Supplementary figures and images for: Filter paper performance in PCR for cutaneous leishmaniasis diagnosis
Source: Rev Soc Bras Med Trop. 2020 Dec 21;54:e00472020. doi: 10.1590/0037-8682-0047-2020 (PMC7747818; doi:10.1590/0037-8682-0047-2020)

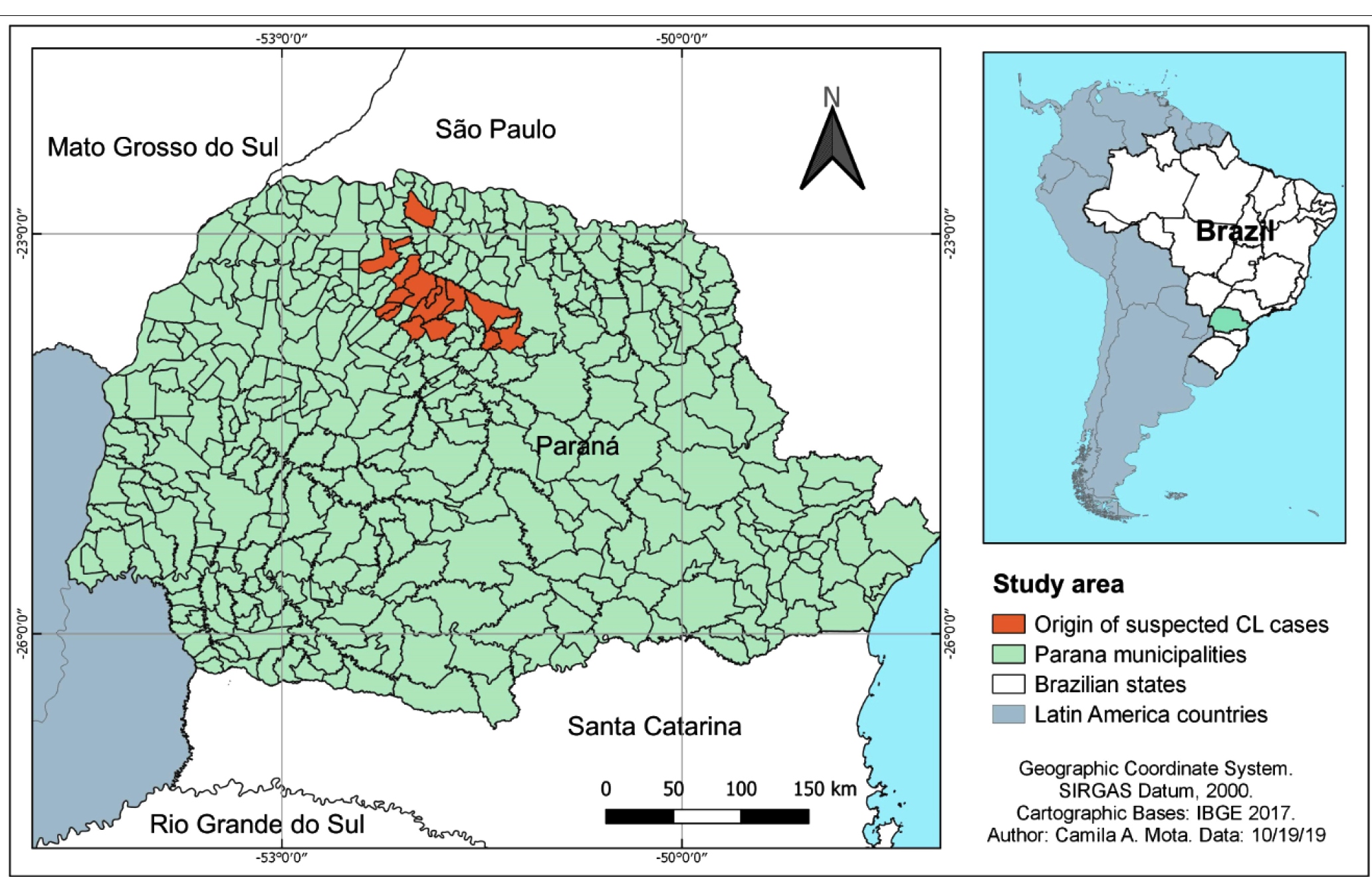

Supplement: Supplementary file 1 [file 1678-9849-rsbmt-54-e00472020-suppl1.jpg]
